# An integrated care program to prevent work disability due to chronic low back pain: a process evaluation within a randomized controlled trial

**DOI:** 10.1186/1471-2474-10-147

**Published:** 2009-11-30

**Authors:** Ludeke C Lambeek, Willem van Mechelen, Peter C Buijs, Patrick Loisel, Johannes R Anema

**Affiliations:** 1 EMGO Institute for Health and Care Research, Department of Public and Occupational Health, VU University Medical Center, Amsterdam, the Netherlands; 2 Body@Work, Research Center Physical Activity, Work and Health, TNO, VU University Medical Center, Amsterdam, the Netherlands; 3 Research Center for Insurance Medicine, AMC-UWV-VUmc, Amsterdam, the Netherlands; 4 University of Toronto, Toronto, Canada

## Abstract

**Background:**

In the past decade, a considerable amount of research has been carried out to evaluate the effectiveness of innovative low back pain (LBP) interventions. Although some interventions proved to be effective, they are not always applied in daily practice. To successfully implement an innovative program it is important to identify barriers and facilitators in order to change practice routine. Because usual care is not directly aimed at return to work (RTW), we evaluated an integrated care program, combining a patient-directed and a workplace-directed intervention provided by a multidisciplinary team, including a clinical occupational physician to reduce occupational disability in chronic LBP patients. The aims of this study were to describe the feasibility of the implementation of the integrated care program, to assess the satisfaction and expectations of the involved stakeholders and to describe the needs for improvement of the program.

**Methods:**

Eligible for this study were patients who had been on sick leave due to chronic LBP. Data were collected from the patients, their supervisors and the involved health care professionals, by means of questionnaires and structured charts, during 3-month follow-up. Implementation, satisfaction and expectations were investigated.

**Results:**

Of the 40 patients who were eligible to participate in the integrated care program, 37 patients, their supervisors and the health care professionals actually participated in the intervention. Adherence to the integrated care program was in accordance with the protocol, and the patients, their supervisors and the health care professionals were (very) satisfied with the program. The role of the clinical occupational physician was of additional value in the RTW process. Time-investment was the only barrier for implementation reported by the multidisciplinary team.

**Conclusion:**

The implementation of this program will not be influenced by any flaws in its application that are related to the program itself, or to the adherence of patients with chronic LBP and their health care professionals.

This program is promising in terms of feasibility, satisfaction and compliance of the patients, their supervisors and the health care professionals. Before implementation on a wider scale, the communication and the information technology of the program should be improved.

**Trials Registration:**

[ISRCTN28478651]

## Background

In the past decade, a considerable amount of research has been carried out to evaluate the effectiveness of interventions to prevent work disability due to low back pain (LBP)[1]. However, in spite of proof of their effectiveness, some interventions are not implemented or applied in practice[2,3]. Therefore, certain questions arise: Why are many innovative and effective interventions not implemented in practice? Why is there a gap between knowledge and practice? The answer to these questions could be that, so far, relatively little attention has been paid to the feasibility of new interventions[1,4], or that it is difficult to implement an innovation when various different stakeholders are involved[5]. It is not only important to implement effective interventions, but it is also important to ensure that they are implemented properly. Inadequate or incorrect implementation of an intervention has a negative effect on the outcome[6].

To obtain insight into whether patients have received the intended intervention as it was designed, and whether the treatment is feasible in daily practice, a process evaluation must be carried out. A process evaluation can provide information about barriers and facilitators for the implementation of an intervention. Barriers and facilitators can be found at four main levels: the level of the patient, the professional (i.e. all persons involved in the implementation) who adopts the innovation, the characteristics of the innovation itself, or the organization and the environment in which the innovation is implemented [7,8]. A process evaluation can also enable care-providers and policy-makers to determine whether the findings of an intervention study apply to their own specific setting, population or country[9].

The present study describes a process evaluation of an integrated care program for sick-listed patients with chronic LBP. This program was based on return to work (RTW) interventions for sick-listed employees with (sub-) acute LBP [10-12] and consists of a combination of patient-directed and workplace-directed interventions co-ordinated by an independent clinical occupational physician, in close collaboration with other involved stakeholders. The bio-psychosocial model of pain and disability was used as the theoretical framework for this study[13]. Within this framework, (work) disability due to LBP is a result of human functioning influenced by biomedical factors (red flags), psychological factors (yellow flags), workplace factors (blue flags) and health care and compensation system factors (black flags) [13-15]. Integrated care for patients with chronic LBP consists of clinical interventions, if needed (red flags), graded activity as a cognitive behavioural intervention aimed at fear avoidance beliefs (yellow flags), a work(place) intervention encouraging the stakeholders to reduce barriers at the workplace (blue flags), and finally, occupational health care integrated into mainstream health care to reduce system barriers (black flags). The main aim of the treatment is to restore human functioning in private and working life, and not to reduce the pain[16]. Details about the content of the program have been published elsewhere[17].

The research questions addressed in this study were: (1) Is it feasible to implement the program according to the protocol?; (2) How do patients, their supervisors and health care professionals evaluate the program?; and (3) What needs to be improved in the program?

## Methods

This process evaluation was carried out as part of a randomized controlled trial (RCT) on the effectiveness of an integrated care program for sick-listed patients with chronic low back pain, the BRIDGE study. The Medical Ethics Committees of the participating hospitals (the VU University Medical Centre, the Slotervaart Hospital, the Amstelland Hospital, the Onze Lieve Vrouwe Gasthuis, all based in Amsterdam, and the Spaarne Hospital based in Hoofddorp) approved the study protocol and all participants gave written informed consent.

### Subjects

Patients between 18-65 years of age, suffering from LBP for at least 12 weeks, with paid work (employed or self-employed) for at least 8 hours per week, and sick-listed were eligible for participation. Excluded were patients with specific LBP or non-specific LBP of less than 12 weeks duration, with cardiovascular pathology or psychiatric pathology, with any type of juridical conflict at work and/or unable to complete questionnaires in the Dutch language. Detailed information about the recruitment procedure has been published elsewhere[17].

### Health care professionals

To provide the integrated care we recruited two clinical occupational physicians and three occupational therapists working in one university hospital, and twenty physical therapists working in ten practices. They all participated in a 2-day training program during which they received information about the study and treated simulated cases. Medical specialists working in 5 hospitals (mainly in the departments of neurology and orthopaedics) were informed about the program, and asked to refer their patients to the study. The primary care physicians of each patient (occupational physician and general practitioner) were informed after the patient had been enrolled in the study.

### Intervention

The overall aim of the integrated care program was to restore occupational functioning and achieve a full sustainable return to own or equal work. Its aim was not to reduce pain. The integrated care was provided by a multidisciplinary team consisting of a clinical occupational physician, an occupational therapist, a physical therapist and other health care professionals such as the patient's primary physicians (general practitioner and occupational physician) and their medical specialist. The clinical occupational physician, was based in a hospital and was responsible for the planning and the co-ordination of the care and (facilitating) communication with the other health care professionals and setting a proposed date for full RTW, in mutual agreement with the patient and the patient's occupational physician. The communication between the team members consisted of telephone calls, mail, coded e-mails, and a conference call every three weeks to discuss the progress of the patient's RTW.

The integrated care program consisted of a work(place) intervention, based on participatory ergonomics, and a graded activity program based on cognitive behavioural principles. The work(place) intervention consisted of 3 meetings (occupational therapist with the patient, occupational therapist with the patient's supervisor and the occupational therapist, the patient and their supervisor together) that focused on identifying and prioritizing obstacles and solutions and achieving consensus between the patient and their supervisor with regard to work adjustments to facilitate RTW. The graded activity program consisted of maximal 26 sessions during a period of 3 months. This program was an individually tailored (to the work situation) exercise program, in which the physical therapist teaches the patient that it is safe to move while increasing the level of physical activity. The focus of the graded activity was to restore occupational functioning capacity in order to achieve RTW. Details about the integrated care program itself have been published elsewhere[17].

### Data-collection

The data for this process evaluation were collected at baseline and during the 3-month follow up, mainly by means of questionnaires, but also from structured charts and a database. Data were collected from: 1) the patient, 2) the clinical occupational physician, the occupational therapist and the physical therapist, and 3) the patient's primary care physicians (general practitioner and occupational physician) and their supervisor who had been involved in the program.

### Outcome measures

#### Reach/participation

The number and representativeness of the patients, their supervisors, the multidisciplinary team and the other health care professionals who participated in this study were registered, as well as reasons for non-participation.

#### Implementation of the integrated care program according to the protocol

In order to determine whether the integrated care program was implemented according to the protocol, we evaluated for each participant: 1) the timeline of the implementation process (start, duration and sessions), and 2) the content of the program (degree to which the main elements of the program were applied). The content of the work(place) intervention was assessed by means of a structured chart, on which the barriers for RTW, the solutions, and the RTW plan were documented. All obstacles and solutions for RTW were classified according to the ergonomic abstracts classification scheme and the definition of work organization in the National Occupational Research Agenda of the National Institute for Occupational Safety and Health[18]. The classification categories were: workplace design; work design and organization (tasks, schedules, communication, training, management style, use of support, organizational characteristics); work environment; and task-related factors (mental workload, physical workload and person-related stress). When the integrated care program had been completed (maximum duration of 3 months), an implementation questionnaire, focusing on barriers and facilitators for implementation of the intervention, was sent to the members of the multidisciplinary team and to the patient's general practitioner, the patient's occupational physician and the patient's supervisor [19,20].

#### Expectations for RTW and satisfaction with the integrated care program

Opinions about satisfaction after participation in the integrated care program, perceived usefulness of the intervention, and expectations for RTW (and symptom recovery) were requested from all stakeholders in the 3-month follow-up questionnaire. Whether patients felt that they had been taken seriously by the members of the multidisciplinary team was measured with the short version of the Patient Satisfaction with Occupational Health Services questionnaire[19], based on a 5-point scale ranging from no agreement to full agreement.

#### Barriers and facilitators for the implementation of the integrated care program

To implement an intervention properly, it is important to be aware of the barriers and facilitators for practical application of the intervention. Therefore, the health care professionals were asked to give their opinion about the applicability of the integrated care program.

### Data-analysis

Baseline and outcome variables were analyzed by means of descriptive statistics, such as frequencies, measures of central tendency and dispersion. Excel 2003 and SPSS version 15.0 were used for the descriptive and statistical analyses. The identified barriers for RTW and the solutions in the work(place) intervention charts were classified by two researchers independently, according to the 'Ergonomics Abstracts' classification scheme [18]. If there was a difference of opinion, consensus was achieved by consulting a third researcher.

## Results

### Reach/participation

#### Patients

**Figure 1:**
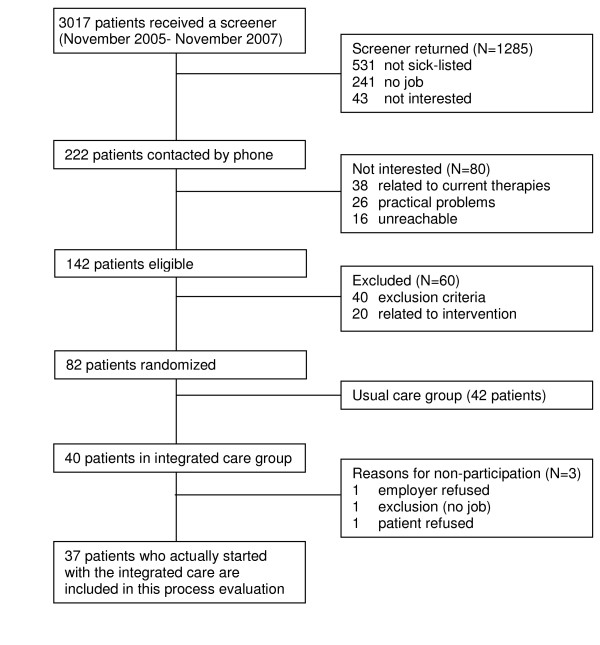
**Flow diagram of patients in the BRIDGE study, including reasons for non-participation**.

Figure 1 shows the flow diagram of patients in the study, including reasons for non-participation. Between November 2005 and April 2007, approximately 3000 screening questionnaires were sent to patients who had visited a medical specialist because of LBP (45.3% male; mean age of 47.3 years). The average response rate was 42%. Based on the screening questionnaires that were returned, 222 patients were contacted by telephone for participation. The main reasons for non-eligibility were not being on sick-leave (41%), and/or not having a job (19%). Of the 222 patients who were contacted by phone, 140 were unwilling to participate or were unable to participate for other reasons. The main reasons for non-participation were related to exclusion criteria (temporary job, no informed consent, insufficient command of Dutch language) (n = 40) and current treatment (satisfied with current treatment, no approval from current health care professional) (N = 38). Finally, 82 eligible patients were randomized. Of the patients who were randomized to the integrated care group (n = 40), 3 did not start to participate in the intervention at all, and 9 started only partially. Reasons for not starting either the graded activity program or the work(place) intervention were related to satisfaction with current treatment (n = 4), other physical complaint (N = 1), the employer's refusal (n = 3) and RTW (n = 1). Data on the 37 patients who finally participated were included in the analyses. The baseline characteristics of these patients are shown in Table 1.

**Table 1:** Baseline characteristics of the patients sick-listed due to chronic low back pain (N = 37)

Patient characteristics	
Age (mean ± sd years)	46.3 ± 7.7
Male (%)	59.4
Sick-leave duration at randomization (mean ± sd days)	136.0 ± 114.0

**Low back pain-related characteristics**	

Diagnosis by medical specialist (%)	
Lumbar disc displacement without myelopathy	52.5
Lumbago	10.0
Sciatica	10.0
Back pain with radiation, unspecified	10.0
Lumbosacral spondylosis without myelopathy	7.5
Spinal stenosis, lumbar region	7.5
Spondylosis of unspecified site without myelopathy	2.5
Pain intensity (1-10 score) (mean ± sd)	6.0 ± 2.3
Functional disability (0-24 score) (mean ± sd)	15.3 ± 4.8
Referred from neurology (%)	79.0

**Occupation-related characteristics**	

Type of work (%)	
Physically demanding work	61.5
Mentally demanding work	38.5
Work sector (%)	
Education	5.4
Construction industry	18.9
Transport and communications	16.3
Health care and public welfare	21.6
Business and financial services	29.7
Government, public safety and security	8.1

#### Multidisciplinary team and other health care professionals

Two clinical occupational physicians, three occupational therapists and twenty physical therapists were invited to participate in the study. They all responded positively and completed the training program. Three of the twenty physical therapists treated only one patient, and therefore their experience with the protocol was considered to be insufficient. The primary care physicians of each patient (their own general practitioner and/or occupational physician) were invited to co-operate after the patient had given informed consent, and all were willing to do so. Twelve departments in five hospitals were also asked to cooperate. The department of neurology in one hospital and one neurologist from another hospital were unwilling to co-operate. The main reason given was that they were not willing to ask patients to participate in the study, due to the extra workload or for reasons of confidentiality.

### Implementation of the integrated care program according to the protocol

The rate of response to the questionnaires was as follows: patients, physical therapists and occupational therapists 100%, supervisors 75% (21/28), general practitioners 72.5% (16/22), and occupational physicians 85% (24/28).

### Timeline of the program

Table 2 shows the timeline (start, duration and number of sessions) of the components of the program. The (median) start of the integrated care was according to the protocol, and the total duration of the clinical occupational physician protocol and the graded activity program was within the range of the protocol. The median time-investment for the work(place) intervention was 9 hours and 20 minutes (interquartile range: 7.7-11.4 hrs), including the time needed for travelling, reporting, administration and organisation of the work(place) intervention. The three meetings (occupational therapist with the patient; with their supervisor; and with the patient and their supervisor together) had a median duration of 4 hours and 45 minutes.

**Table 2:** Time-line of the components of the integrated care program

		*Start after inclusion (days)* *according to*			*Duration of intervention (days)* *according to*			*Number of sessions* *according to*		
		**protocol** **(max)**	**study** **(median, [IQR])**		**protocol** **(max)**	**study** **(median, [IQR])**		**protocol** **(max)**	**study** **(median, [IQR])**	

***Clinical occupational physician protocol***		7	6	[4.0-7.5]	*84*	56	[32.5-73.0]	3	2	
***Contact OP***		9	6	[4.0-8.0]	-	-		-	-	
***Contact MS, GP***		9	6	[4.3-7.8]	-	-		-	-	
***Contact PT, OT***		11	8	[6.0-11.0]	-	-		-	-	

***Graded activity protocol***		14	15	[13.0-28.0]	*84*	62	[36.0-82.0]	26	17	[12.0-24.0]

***Work(place) intervention***		21	25	[19.8-29.3]	*28*	49	[28.5-75.0]	3	2	[2.0-3.0]

Max: maximum; IQR: interquartile range; OP: occupational physician; MS: medical specialist;

GP: general practitioner, PT: physical therapist; OT: occupational therapist.

### Content of the program

At the start of the program the clinical occupational physician had contacted the occupational physician, the physical therapist and occupational therapist for respectively 65%, 100% and 100% of the patients. When providing the integrated care, the clinical occupational physician communicated (in addition to communication times) with the occupational therapist and physical therapist by mail or phone (respectively 25% and 50% of the cases) to discuss the patient's progress and/or to adapt the treatment. The agreed RTW date was changed three times (3/37). The date for full RTW, set in mutual agreement between the clinical occupational physician, the patient and the patient's occupational physician, was achieved in 72% of the cases. Only a few conference calls, involving all the health care professionals, took place (5/37).

**Figure 2:**
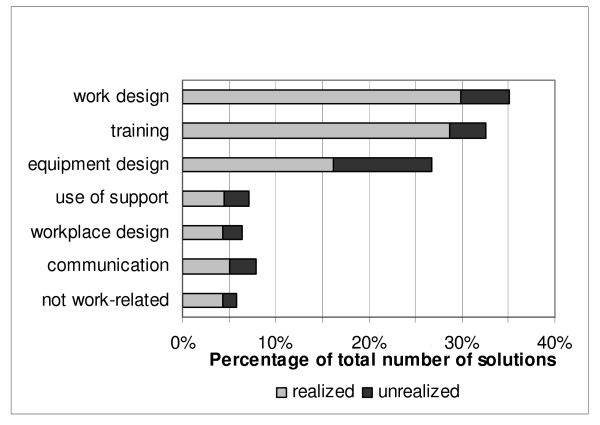
**Proportion of realized and non-realized solutions in the total number of solutions**.

The three individually chosen exercises related to problems in the work situation mainly concerned sitting (23%), lifting (21%) and standing (15%). According to the physical therapists, the exercise goals set in the graded activity treatment plan were achieved in 77% of the patients (24/31). The work(place) intervention identified a total of 165 barriers and 324 solutions for RTW. Most of the barriers were related to physical workload (36.4%) and work design (25.5%). The solutions, on the other hand, concerned changes in work design (25.3%), training (22.2%) and changes in equipment design (20.7%). Table 3 presents some examples of identified barriers and proposed solutions identified during the work(place) intervention. Figure 2 shows that most of the solutions (72%) could be realized in the short-term (within 3 months). Of the 324 solutions, 28% had not been realized at the time of evaluation. In 16% of the solutions the reason for non-implementation was unknown, and in 9% of the cases another solution had been found. According to the occupational therapists, 80% of the patients had had sufficient say in the work(place) intervention process. The other 20% had not, for the following reasons: a disturbed relationship between the patient/supervisor (n = 2); reorganization in the company (n = 1), no supervisory support for participation in the program (n = 2), or no agreement with regard to solutions (n = 2).

**Table 3:** Examples of identified barriers for RTW and the proposed solutions

Example	Barriers identified	Proposed solution(s)
1	Incorrect posture during telephone conversation	Use hand- free telephone

2	Painful eyes because of insufficient light at workplace	Provide a desk lamp

3	Absence of lift to move equipment	1. Train physical capacity
		2. Ask co-workers to help
		3. Use lifting resources

### Experiences, usefulness and satisfaction

#### Patients

Overall, the patients were very satisfied with the guidance provided by the clinical occupational physician, the occupational therapist and the physical therapist, rated with scores of respectively 7.9, 7.9 and 8.6 (scale 0-10; 10 indicating maximum satisfaction). They reported that participation in the graded activity program (23/31) and work(place) intervention (18/34) had had a beneficial effect on RTW. With regard to the question about the usefulness of participation in the graded activity program and the work(place) intervention, the patients were positive about both (respectively 80.6% and 64.5% of the patients). Even though the graded activity program focused on RTW, two-thirds of the patients indicated that participation in the program had resulted in less LBP. The patients also reported that the graded activity program had contributed to their knowledge about how to prevent LBP (20/31), how to reduce back pain (23/31) and how to achieve full job performance (21/31).

#### The multidisciplinary team and other health care professionals involved

**Figure 3:**
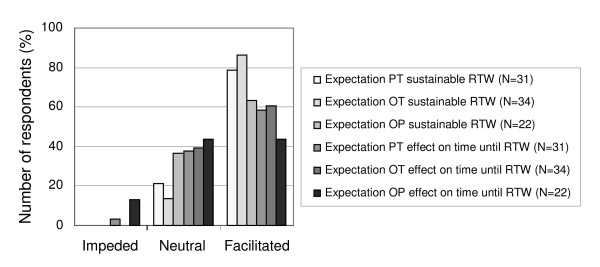
**Expectations of sustainable RTW and effect on time until RTW rated by the physical therapists, occupational therapists and occupational physicians as a percentage of the number of respondents**. PT: physical therapist; OT: occupational therapist; OP: occupational physician; RTW: return to work.

In general, the physical therapists and the occupational therapists were satisfied with the process of the program (respectively 64% and 56%). They were especially satisfied with regard to the use of the communication charts, the collaboration between the multidisciplinary team members, and the tailoring of the treatment plan to the patient. They were also satisfied with the conference calls, although these had only been made a few times. The main reason for the low frequency of these calls was the difficulties that were encountered in organizing a conference call. The patient's occupational physicians were also satisfied with the process of the program (52%). The expectations of the patient's occupational physicians, physical therapists and occupational therapists with regard to the contribution of the integrated care to sustainable RTW and time until RTW were positive (see Figure 3).

#### Supervisors

The majority (57%) of the supervisors were (very) satisfied with the guidance provided by the clinical occupational physicians and the occupational therapists and they were satisfied with the chosen work adaptations. According to the supervisors (n = 28) the most important aspects of a successful work(place) intervention were the employee's expectations of the effectiveness of the integrated care (82%) and the employee's trust in their supervisor (82%). The supervisors noted that they had all participated sufficiently in finding solutions for the work adaptations, that the chosen solutions encouraged RTW (85%), and that none of the work(place) interventions had caused a delay in RTW.

### Barriers and facilitators for implementation

After each integrated care program, the health care professionals evaluated the process of the care by rating various implementation factors as impeding, neutral or facilitating. The most important factors that were positively related to the process of the care were motivation of the patients for RTW, the commitment, and the compliance of the patients and trust of the patient in their supervisor. The factors that were negatively related to the process of the care, according to the health care professionals, were lack of commitment of the patient supervisor, reduced physical capacity of the patient, and the duration of the care. In general, factors related to communication were mainly rated as neutral.

After all the patients had completed the integrated care program, the health care professionals were asked to evaluate the implementation of the program in general. According to all members of the multidisciplinary team, application of the program was appropriate when there were problems in communication with the employer, when there were irrational cognitions of bodily movement, and when patients showed chronic pain behaviour. Application of the program was not recommended if the patient had any juridical conflict with the employer, lacked motivation, had uncomplicated LBP, or was physically very fit. Over 80% of the members of the multidisciplinary team (n = 18) stated that the involvement of the clinical occupational physician was of additional value. The presence of various perceived barriers according to the multidisciplinary team is shown in Table 4. With the exception of time-investment, all characteristics of the program were rated positively, and therefore positively influenced the implementation of the program.

**Table 4:** Perceived barriers for implementation of the intervention by the multidisciplinary team (N = 18)

Level	Factor	No barrier perceived (N)	Undecided (N)	Barrier perceived (N)
**Innovation**	Scientific basis	18	0	0
	Flexibility	13	3	2
	Complexity	11	5	2
	Compatibility	13	2	3
	Time-investment	5	6	7

**Health care Professionals**	Attitude	18	0	0
	Knowledge	17	0	1
	Perceived advantage	15	1	2
	Expertise	18	0	0

**Context**	Resistance of patients	14	2	2
	Resistance of employers/other health care professionals	14	3	1

## Discussion

The aim of this paper was to evaluate the implementation process and experiences with an integrated care program, as reported by patients with chronic LBP, their supervisors and their health care professionals. The main results indicated that, in general, the program was implemented according to the protocol, and overall satisfaction with the program was rated high by the patients, their supervisors, and the health care professionals.

### Comparison with other studies

The implementation of a graded activity program and/or a (work)place intervention had already been carried out for patients with (sub-)acute LBP who were on sort-term sick-leave[2,3,6]. Comparison of the type of obstacles and solutions for RTW identified in the present study (patients with chronic LBP and their supervisors) with the results of studies in which patients with (sub-)acute LBP were involved, showed that these obstacles and solutions were comparable[2,3]. Implementation of the solutions, on the other hand, differ considerably: 72% in the present study, compared to 50% in patients with (sub-)acute LBP[2,3]. The reasons for this difference could be related to the difference in motivation between patients with sub-acute and chronic LBP. Patients with chronic LBP and their supervisors could have been more motivated to implement the solutions, because these patients experienced it as a last opportunity to deal with their complaints[21]. Their supervisors might be more motivated to implement solutions, because it is known that the longer an employee (with chronic LBP) is sick-listed, the less likely it is that employee will return to work[22,23].

Compliance with the graded activity program in the present study was high, compared to the compliance of patients with (sub-)acute LBP in the Steenstra study (respectively 80% compared to 66%)[6]. These differences could also be related to differences in the study population. Patients with chronic LBP are more motivated to comply with the graded activity program because they have long medical histories, with frequent visits to various health care providers, which were mostly related to pain reduction and did not lead to long-term functional improvement. They experienced participation in this study as a last hope of solving their problems[21].

### Strengths and limitations of this study

Since all stakeholders have different interests in the field of work disability, a strength of our study was that we evaluated the process, expectations for RTW, satisfaction, and compliance with the integrated care program from the perspective of all the different stakeholders (the patients, their supervisors and the health care professionals) involved in the implementation of the integrated care program[24,25]. In particular evaluation from the perspective of the patient's supervisors is of great value because the supervisor has a key role in the prevention of work disability[25]. Another strength of this study is the triangulation of research methodology, which makes the results (more) reliable. The experiences of the stakeholders with the integrated care program were investigated in a quantitative manner in our study, and in a qualitative manner by Buijs et al[21]. The results of the two studies are comparable.

There are also some methodological weaknesses in our study. First of all, selection bias might have occurred because we included only patients who were motivated to participate. Nevertheless, we do not expect that this will have had any influence on the results because Buijs et al. showed that the intention to participate differed among the participants[21]. The motivation of some patients to participate in the study was related to pain reduction even though the primary goal of the integrated care program was RTW. In spite of differences in motivation at the start of the program, most of the participants were positive about the intervention and the drop-out rate was relatively low. Therefore, we think that the integrated care program succeeded in emphasizing the importance of RTW, instead of focusing on pain reduction. Secondly, some of the health care professionals in the multidisciplinary team (clinical occupational physicians, physical therapists, occupational therapists) were self-selected, and might therefore have been more motivated than others. However, to test the feasibility of the implementation of an intervention on a broader scale, it is important to first evaluate the experiences of motivated people on a small scale[26].

### Practical implications

By adding a clinical occupational physician to the multidisciplinary team we tried to overcome communication problems between the stakeholders. The results of this study showed that the patients and the health care professionals were satisfied with the inclusion of a clinical occupational physician, but there is still room for improvement. The communication between health care professionals can be improved by introducing a computerized support system instead of (separate) databases, and making use of coded e-mails instead of conference calls. For broader implementation of the integrated care program it is also essential to pay more attention to barriers related to time-investment.

Besides these points of improvement, we do not assume that implementation will be difficult, because in our study the costs of both interventions (the graded activity program and the work(place) intervention) were covered by the patient's health insurance.

## Conclusion

Based on this pilot study, wide-scale application and implementation of the integrated care program is feasible, although more attention must be paid to improving communication between the health care professionals. According to the involved stakeholders, the innovative role of the clinical occupational physician was of additional value in the RTW process. The compliance and satisfaction of patients, health care professionals and patient's supervisor with the program was high so the feasibility of implementing this innovative intervention program on a broader scale is promising.

## Abbreviations

RTW: Return to work; LBP: Low back pain; RCT: Randomized controlled trial; SD: standard deviation.

## Declaration of competing interests

The authors declare that they have no competing interests.

## Statement of authors

We confirm that all details of the patients have been omitted or in such a way that the patients cannot be identified through any of the descriptions.

## Authors' contributions

LCL was involved in the development of the study design, responsible for the general co-ordination of the study, the implementation of the integrated care program, carried out the data-collection and participated in writing the paper. JRA was involved in the development of the study design, was responsible for the general co-ordination of the study, the implementation of the integrated care program and participated in writing the paper. PB, WvM and PL were involved in the development of the study design and contributed to writing the paper. All authors read and approved the final version of the manuscript.

## Pre-publication history

The pre-publication history for this paper can be accessed here:

http://www.biomedcentral.com/1471-2474/10/147/prepub
